# Extracellular matrix alignment dictates the organization of focal adhesions and directs uniaxial cell migration

**DOI:** 10.1063/1.5052239

**Published:** 2018-12-26

**Authors:** William Y. Wang, Alexander T. Pearson, Matthew L. Kutys, Colin K. Choi, Michele A. Wozniak, Brendon M. Baker, Christopher S. Chen

**Affiliations:** 1 Department of Biomedical Engineering, University of Michigan, Ann Arbor, Michigan 48109, USA; 2 Section of Hematology/Oncology, Department of Medicine, The University of Chicago, Chicago, Illinois 60637, USA; 3 The Biological Design Center and Department of Biomedical Engineering, Boston University, Boston, Massachusetts 02215, USA; 4 Wyss Institute for Biologically Inspired Engineering, Harvard University, Boston, Massachusetts 02115, USA; 5 Department of Bioengineering, University of Pennsylvania, Philadelphia, Pennsylvania 19104, USA

## Abstract

Physical features of the extracellular matrix (ECM) heavily influence cell migration strategies and efficiency. Migration in and on fibrous ECMs is of significant physiologic importance, but limitations in the ability to experimentally define the diameter, density, and alignment of native ECMs *in vitro* have hampered our understanding of how these properties affect this basic cell function. Here, we designed a high-throughput *in vitro* platform that models fibrous ECM as collections of lines of cell-adhesive fibronectin on a flat surface to eliminate effects of dimensionality and topography. Using a microcontact printing approach to orthogonally vary line alignment, density, and size, we determined each factor's individual influence on NIH3T3 fibroblast migration. High content imaging and statistical analyses revealed that ECM alignment is the most critical parameter in influencing cell morphology, polarization, and migratory behavior. Specifically, increasing ECM alignment led cells to adopt an elongated uniaxial morphology and migrate with enhanced speed and persistence. Intriguingly, migration speeds were tightly correlated with the organization of focal adhesions, where cells with the most aligned adhesions migrated fastest. Highly organized focal adhesions and associated actin stress fibers appeared to define the number and location of protrusive fronts, suggesting that ECM alignment influences active Rac1 localization. Utilizing a novel microcontact-printing approach that lacks confounding influences of substrate dimensionality, mechanics, or differences in the adhesive area, this work highlights the effect of ECM alignment on orchestrating the cytoskeletal machinery that governs directed uniaxial cell migration.

## INTRODUCTION

Directed cell migration plays a fundamental role in numerous physiologic and pathologic processes, where biochemical and physical cues from the extracellular matrix (ECM) guide cells to their target destinations.^1–3^ In wound healing, gradients of chemokines recruit macrophages to wound sites to initiate the tissue repair process.^4^ Similarly, during the progression of most solid cancers, gradients of growth factors produced by the tumor mass direct the invasion of endothelial cells required for angiogenesis and subsequent tumor growth.^5^ While soluble cues clearly are important in mediating directed cell migration, physical features of the microenvironment have more recently been implicated in this process. For example, early work demonstrated that contact guidance produced by the orientation and spatial restriction of fibronectin fibrils during amphibian gastrulation direct mesodermal cell migration from the blastopore to the animal pole.^6^ In the context of breast cancer, Provenzano *et al.* described how the collagenous stroma undergoes marked reorganization, resulting in radially aligned collagen tracts emanating from the tumor. This organization in turn appears to facilitate cancer cell escape from the primary tumor.^7,8^ In both of these rather diverse processes, directional cues influencing cell migration arise from the fibrous ECM that cells negotiate during migration.

Given its ubiquity throughout the body, type I collagen gels have been widely used as a physiologically representative ECM model for *in vitro* cell migration studies.^9–14^ These studies further implicate physical attributes of the ECM such as alignment, density, diameter, and stiffness as integral factors in directing cell migration. However, identifying the individual contribution of these factors proves to be quite challenging in natural materials such as collagen gels. For example, increasing the concentration of a collagen gel concurrently increases fibril density, matrix stiffness, and cell-adhesive ligand density while decreasing the pore size. Thus, bioengineered *in vitro* models that simplify the complexity of natural ECM and can decouple confounding factors have helped deepen our understanding of how the physical properties of the ECM regulate cell migration.^15–19^

In particular, cell migration on micropatterned lines of adhesive ECM proteins has been suggested to recapitulate migration observed within *in vivo* 3D microenvironments composed of highly aligned fibers.^20–22^ NIH3T3 fibroblasts migrating on single lines of patterned adhesive ECM proteins, termed “1D migration,” exhibit a uniaxial cell morphology and undergo directed migration with similar speed as in 3D cell-derived matrices, whereas cells undergoing unconstrained migration on unpatterned 2D surfaces do not.^20^ Similarly, 1D micropatterned substrates have also been used to reconstitute macrophage-tumor cell interactions, validating intravital observations in highly metastatic patient-derived orthotopic mammary tumors.^21^ These and other studies^23^ implicate the dimensionality of the ECM substratum in dictating the cell migration phenotype and suggest that 1D lines of ECM recapitulate key aspects of cell migration observed in 3D fibrillar tissue settings; however, orthogonal control over the adhesive area proved to be difficult in these studies. Furthermore, in addition to dimensionality, there may be other aspects of the ECM which are important in regulating the migration phenotype. Given that many mesenchymal tissues contain adhesive fibers of ECM proteins such as fibronectin and collagen, we wondered how restricting cell adhesion to linear tracks of ECM influences migration, and furthermore, if the geometry and organization of these patterns, independent of substrate dimensionality and mechanics, could dictate the cell migration mode.

To answer these questions, we designed a microcontact printing-based ECM parameter screening tool where we modeled fibrous ECM as arrays of cell-adhesive micron-scale linear elements. We then varied over graded steps three pertinent features reflecting the distribution of adhesive ligands in fibrillary microenvironments (line alignment, density, and width) and examined changes in the morphology, cytoskeletal architecture, and migratory behavior. Using these 2D micropatterned substrates to exclude the influence of substrate dimensionality and mechanics, we found that the alignment of ECM had a dominant effect on cell morphology and migration over other geometric factors, where high alignment induced a uniaxial phenotype and rapid cell migration in a directed fashion. Underlying directed cell migration is the matrix alignment correlated with highly organized focal adhesions (FAs) and actin stress fibers. Members of the Rho family of small GTPases have emerged as prominent players in cell motility, working to spatiotemporally modulate the signaling processes involved in cell adhesion and cytoskeletal dynamics.^79^ In particular, Rac1 is necessary for lamellipodium extension preceding the formation of new adhesions to ECM and has been implicated in directed cell migration, where experimentally decreasing Rac1 activity switches cells from random to directionally persistent migration.^90^ Given the observed influence of matrix alignment on directed cell migration, we further used two distinct molecular approaches to examine Rac1 localization and found that matrix alignment additionally influences the number and location of protrusive edges initiated by active Rac1.

## RESULTS AND DISCUSSION

### Multiparameter ECM screening arrays for high content imaging

**FIG. 1.**
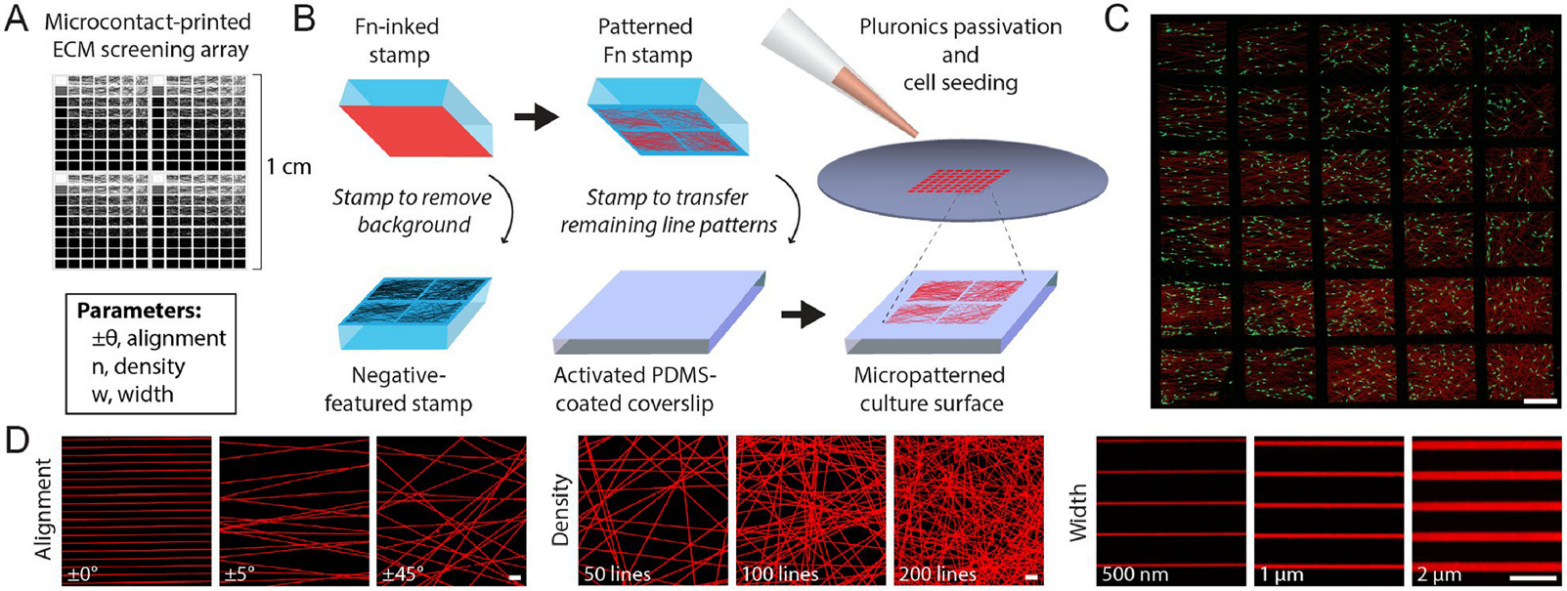
Microcontact-printed ECM screening arrays. (a) Photomask containing 252 individual ECM conditions, each containing a unique randomly generated array of line elements with varying alignments (angular dispersion), line densities, and line widths. (b) Overview of the negative microcontact-printing procedure. Briefly, AlexaFluor555-conjugated fibronectin (Fn555, red) was uniformly coated on flat PDMS stamps and a UV-ozone activated stamp containing the negative of the intended patterns was applied to remove background areas before transferring the remaining areas to the eventual cell culture substrate. (c) Tile-scan fluorescence image of a subsection of the Fn-printed (red) ECM screening array seeded with phalloidin-AlexaFluor488 stained 3T3s (green) (scale bar: 1 mm). (d) Fn555 microcontact-printed patterns demonstrating control over line alignment (left), line density (middle), and line width (right) (scale bars: 10 *μ*m).

Given the ubiquity of micrometer-scale diameter fibrous proteins throughout mammalian extracellular matrices (ECM), we sought to develop a model that captured key aspects of adhesive ligand patterning in fibrous ECMs without confounding factors stemming from substrate dimensionality and mechanics.^15,16,29^ To do so, we simplified fibrous ECMs into two-dimensional (2D) patterns of microcontact-printed fibronectin lines where the angular dispersion (alignment), number (density), and size (width) of these cell-adhesive line elements could be orthogonally varied [Fig. 1(a)]. Building upon previous work,^20^ this microcontact printing-based approach allowed us to explore how the organization of adhesive ECM proteins influenced cell migration, while sidestepping confounding factors such as steric hindrance,^30–34^ the requirement for proteolytic activity,^35–38^ and the influence of substrate mechanics.^36,39–43^ Using custom MATLAB scripts, a prescribed number of initiation points were randomly selected in 2D space, and lines were extended in a direction selected randomly within prescribed bounds. To achieve submicron features, we employed a stepper with 5× optical reduction resulting in silicon masters with SU8 photoresist line features ranging between 0.5 *μ*m and 2 *μ*m in width. In total, 252 unique ECM patterns were generated within a single 1.2 × 1.2 cm^2^ stamp, enabling high-throughput screening over a wide range of adhesive microenvironment parameters using high content tile-scan imaging [Fig. 1(c)].

The desired production of micron-scale features ranging widely in spacing across a single stamp prevented us from using standard “forward” microcontact printing, where the featured stamp is protein inked prior to transfer. Indeed, when conventional “forward” stamping was employed, only patterns with greater than 400 lines/pattern could be successfully generated; at lower line densities, stamp collapse led to protein transfer from non-feature background areas. Thus, we employed an inverse microcontact printing or “stamp off” technique,^44^ whereby a flat PDMS stamp is uniformly inked with adhesive protein and the activated inverse-featured stamp is first applied to remove background areas of protein before transferring the remaining protein pattern to the culture substrate [Fig. 1(b)]. This method enabled the production of nearly all patterns over a range of line alignments (±5°, 15°, 30°, 45°, 60°, and 90°), densities (50, 100, 200, and 400 lines/pattern), and widths (0.5, 0.7, 1.0, and 2.0 *μ*m) [Fig. 1(d)]; arrays with 800 line/pattern at line widths of 1 and 2 *μ*m possessed a larger line area than the total pattern area, resulting in a uniform region of fibronectin. Silicon masters were generated with 0.5 and 2.0 *μ*m thickness photoresist, with 2.0 *μ*m thick features generally leading to more consistent and faithful pattern reproduction. Although linear adhesive patterns below 0.5 *μ*m have been produced previously by other means and may impact focal adhesion growth and dynamics,^45^ the 0.5 *μ*m wide lines achieved here match the limit previously reported for microcontact printing proteins using PDMS.^46^ In summary, we successfully created multi-parametered micropatterned ECM screening arrays to model and study cell migration in fibrous microenvironments.

### Array-wide parameter screen reveals that matrix alignment robustly dictates cell morphology and orientation

**FIG. 2.**
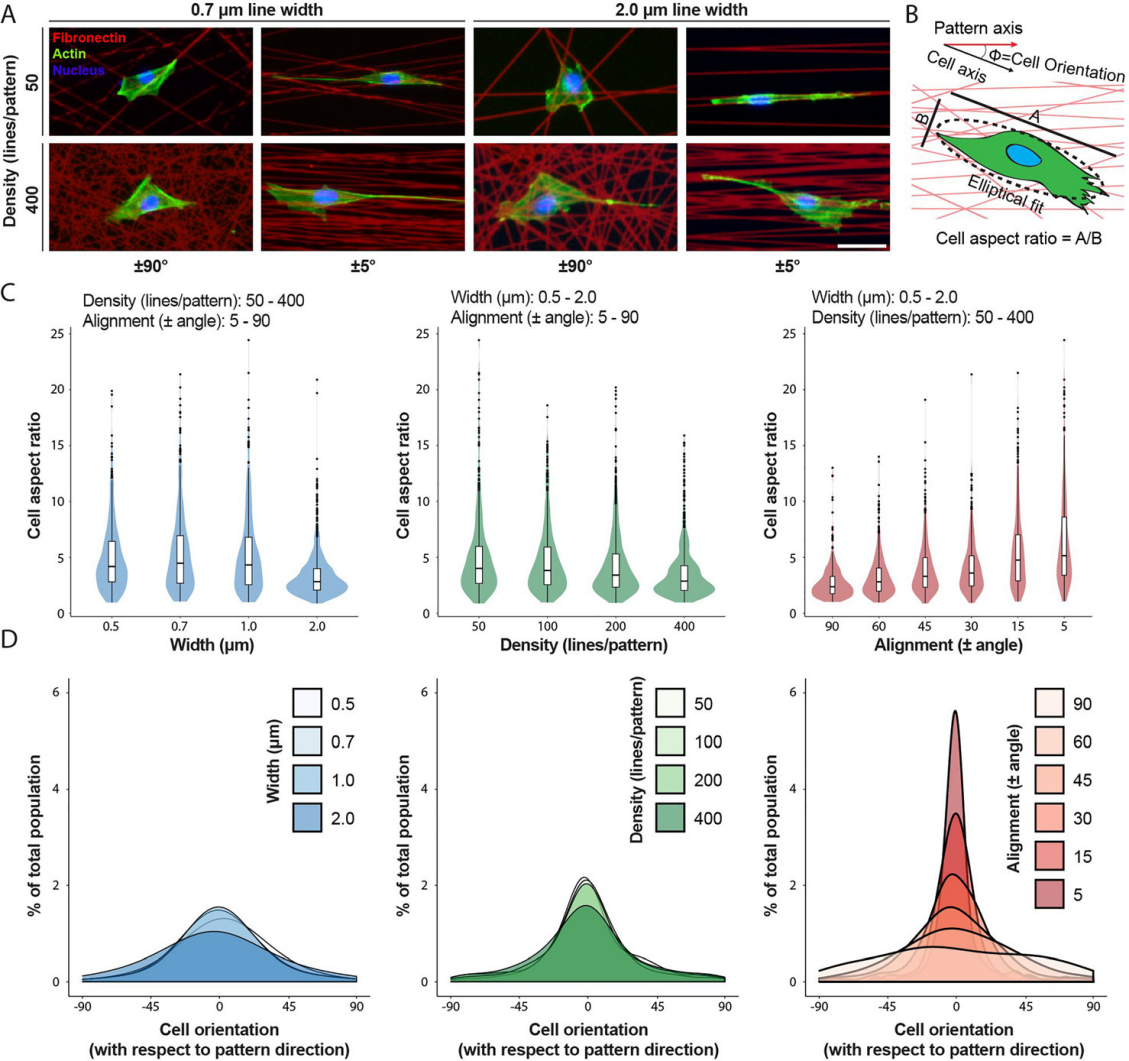
Multiparameter screening of ECM attributes reveals that fiber alignment strongly dictates the cell's aspect ratio and orientation. (a) Representative 3T3 fibroblasts seeded on selected Fn-patterns (red) stained for F-actin (green) and nuclei (blue) with phalloidin-AlexaFluor488 and Hoechst33342, respectively (scale bar: 50 *μ*m). (b) Schematic indicating how the cell aspect ratio and orientation were quantified. (c) Cell aspect ratio as a function of line width (left), line density (middle), and line angular dispersion (right). Data are presented as violin plots demonstrating distribution with overlaid box plots. (d) Histograms of cell major axis orientation with respect to the pattern axis as a function of line width (left), line density (middle), and line angular dispersion (right) (n ≥ 400 cells per condition, total of 2938 cells analyzed).

We next performed an extensive screen across the full array (with the exception of 800 lines/pattern arrays) to examine the effect of varying line alignment, density, and width on cell morphology [Fig. 2(a), supplementary material Fig. 1]. For these and all subsequent studies, we chose NIH3T3s as a model cell type used widely to study how microenvironmental cues influence mesenchymal cell migration.^47,48^ High content tile-scan fluorescence imaging was performed on cells stained for their nucleus and actin cytoskeleton [Fig. 2(a)], with subsequent semi-automated image analysis to extract an assortment of morphometric features. Of the features examined, we immediately identified two factors—cell aspect ratio and orientation—which varied markedly across the array [Fig. 2(b)]. In particular, when analyzing the entire dataset considering each parameter independently, we observed that decreasing line widths, decreasing line densities, and increasing alignment all led to an increase in the cell aspect ratio [Figs. 2(c) and 2(d)]. A high aspect ratio corresponds to a uniaxial cell shape and has been heavily implicated in directed cell migration, where the direction of the cell's long axis (or orientation) defines the direction of migration.^2^

Further statistical analyses were performed to determine the relative strength of the effect of line alignment, density, and width on the cell aspect ratio and variation in cell orientation. When the degree of linear correlation between cell aspect ratio vs. alignment, density, and width was determined, we found that the aspect ratio had the highest correlation with alignment (r^2^ = 0.156, p < 0.0001). The aspect ratio was also independently correlated with density (r^2^ = 0.029, p < 0.0001) and width (r^2^ = 0.089, p < 0.0001). To understand the relationships among alignment, density, and width in explaining the variations in both the aspect ratio and the orientation, we performed multinomial multivariate linear regression model building using a dual-direction selection algorithm from a full interaction model, optimizing for the Akaike information criterion. The model selection proceeded for two rounds and eliminated two interaction terms from the model, without adding back either term. In our final model, alignment, density, width as well as the width by alignment and alignment by density terms were each significant for the multivariate outcome pair of cell orientation and aspect ratio (Table I). The interaction term between alignment and density, for example, implies that at higher alignments, density more potently increases the aspect ratio. We also determined that the variation within cell orientation was significantly associated with alignment (p < 0.0001), but not with density or width. Taken together, the combination of high-throughput screening of matrix conditions and statistical analyses indicates that while line width and the density significantly altered the cell aspect ratio, only the alignment of adhesive ECM tracks strongly influenced cell shape towards a uniaxial phenotype and simultaneously exerted a pronounced effect in orienting cells towards the underlying central axis of pattern alignment. Given the effect of matrix alignment in these and numerous previous studies, our subsequent studies focused on the effect of matrix alignment, maintaining the line width constant at 1 *μ*m.

**TABLE I.** Statistical outcomes from multinomial multivariate linear regression analysis.

Variable	Orientation: response coefficient	Aspect ratio: response coefficient	Multinomial approximate F-statistics	Multinomial approximate p-value
Intercept	0.2780	0.1064	4425.2	<0.001
Width	−5.413 × 10^−4^	−2.551 × 10^−3^	196.4	<0.001
Alignment	0.1374	−8.828 × 10^−2^	293.0	<0.001
Density	−2.662 × 10^−3^	−6.200 × 10^−3^	96.9	<0.001
Width: alignment	−6.455 × 10^−5^	2.754 × 10^−5^	48.8	<0.001
Alignment: density	−1.604 × 10^−4^	5.648 × 10^−5^	15.6	<0.001

### ECM alignment polarizes cells and induces directed cell migration

**FIG. 3.**
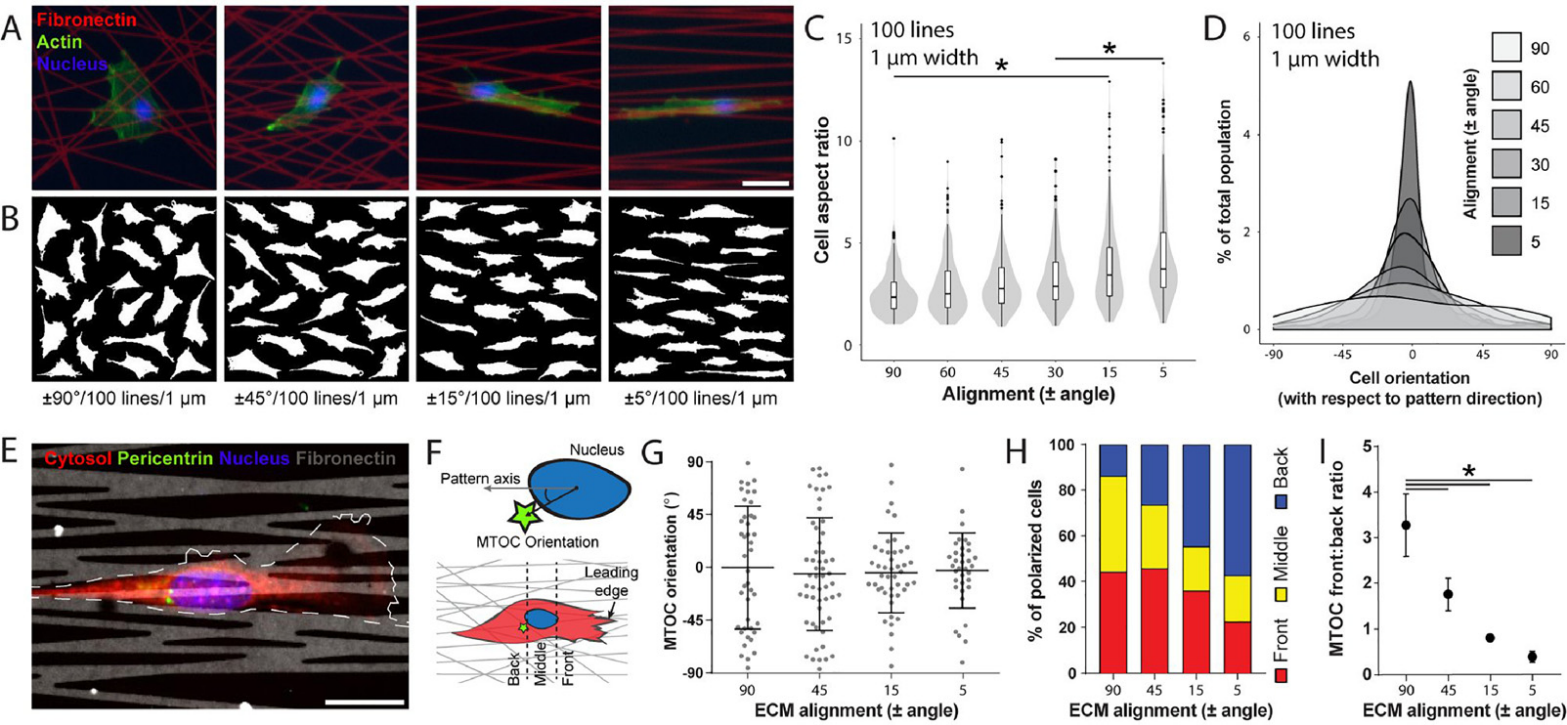
Increasing ECM alignment promotes an elongated uniaxial cell morphology and polarizes the cell. (a) Representative 3T3 fibroblasts seeded on Fn-patterns (red) of varying alignment with fixed line density and width (100 lines per pattern, 1 *μ*m width), stained for F-actin (green) and nuclei (blue) with phalloidin-AlexaFluor488 and Hoechst33342, respectively (scale bar: 50 *μ*m). (b) Cell outlines of 20 representative cells. (c) Aspect ratio and (d) cell orientation (angle between the long axis of the cell and the fiber alignment direction) of 3T3 fibroblasts as a function of ECM alignment, keeping line density constant at 100 lines per pattern and line width at 1 *μ*m width (n ≥ 50 cells per condition, total of 515 cells analyzed). (e) Representative confocal image of a 3T3 fibroblast immunostained for pericentrin to localize the microtubule organizing center (MTOC) (red: cytosol, green: pericentrin, blue: nucleus, and grey: Fn555; scale bar: 25 *μ*m). (f) Schematic indicating how MTOC orientation (angle between MTOC and the pattern axis using the centroid of the cell nucleus as a reference point) and position (front, middle, or back relative to the nucleus and leading edge of the cell) were determined. (g) MTOC orientation as a function of ECM alignment (n ≥ 35 cells per condition, total of 177 cells analyzed). (h) Percentage of all polarized cells with MTOC located in front, in the middle, or rearwards of the nucleus (n ≥ 60 cells per condition, total of 468 cells analyzed). (i) Ratio between cells with MTOC located in front of the nucleus to cells with MTOC located rearwards of the nucleus (* indicates a significant difference with p < 0.05).

The multiple regression parameter screen identified matrix alignment as the strongest factor in dictating cell morphology and orientation. Isolating alignment's individual effect on cell morphology, we held the line density and width constant and examined the cell aspect ratio and orientation (supplementary material Fig. 1). Confirming our initial findings, increasing matrix alignment resulted in an increased cell aspect ratio, causing cells to adopt an elongated morphology and orient preferentially towards the underlying pattern's direction of alignment [Figs. 3(a)–3(d)]. This trend was found to be consistent across all line density conditions examined (data not shown). Gross cell morphology alone, however, does not necessarily indicate cell polarity. Indeed, elongated cells on aligned ECM were occasionally observed to extend bidirectionally without evidence of a dominant leading edge. To better understand the influence of matrix alignment on cell polarization, we performed immunofluorescence staining for pericentrin to identify the microtubule organizing center (MTOC), as previous work suggests that the MTOC's location indicates cell polarity [Fig. 3(e)].^20,49^ We first examined the orientation of the MTOC with respect to the pattern axis [Fig. 3(f)] and observed that increasing matrix alignment induced a higher percentage of cells to position their MTOC along the pattern axis within ±45° of the pattern axis [Fig. 3(g)]. Several reports highlight the importance of nuclear positioning during cell migration,^31,49–52^ and so, we next examined the position of the MTOC relative to the nucleus and leading edge of the cell [Fig. 3(f)]. Interestingly, we found that with increasing alignment, the MTOC preferentially positioned to the rear of the nucleus with respect to the leading edge in polarized and elongated cells [Figs. 3(h)–3(i)]. The position of the MTOC and its role in directed cell migration have been widely debated. The MTOC positions to the front of the nucleus with respect to the leading edge in classical studies performed with scratch wound assays—typically involving a flat 2D substrate with uniform ECM coating.^49,53–55^ In contrast, the MTOC has been observed to favor the rear of the cell in certain cell types, in soft matrices, and in substrates that constrain the cell to 1D micropatterned lines or 3D cell-derived matrices with high matrix alignment.^20,56–58^ Our studies support two possible forms of MTOC positioning and cell polarity as a function of matrix alignment: (1) an aligned uniaxial phenotype previously observed on parallel lines and microgrooves/micropatterned lines occurring on anisotropic ECMs where the MTOC is positioned to the rear of the nucleus and direction of migration and (2) a polarized, but non-uniaxial phenotype that occurs on isotropic ECM (e.g., on non-patterned ECM or on scratch-wound assays) where the MTOC is positioned ahead of the nucleus.^49^ Although an understanding of how ECM properties influence cell polarity remains incomplete, our study clearly demonstrates that the anisotropy of adhesive ECM patterning influences cell shape, intracellular organization, and polarity.

**FIG. 4.**
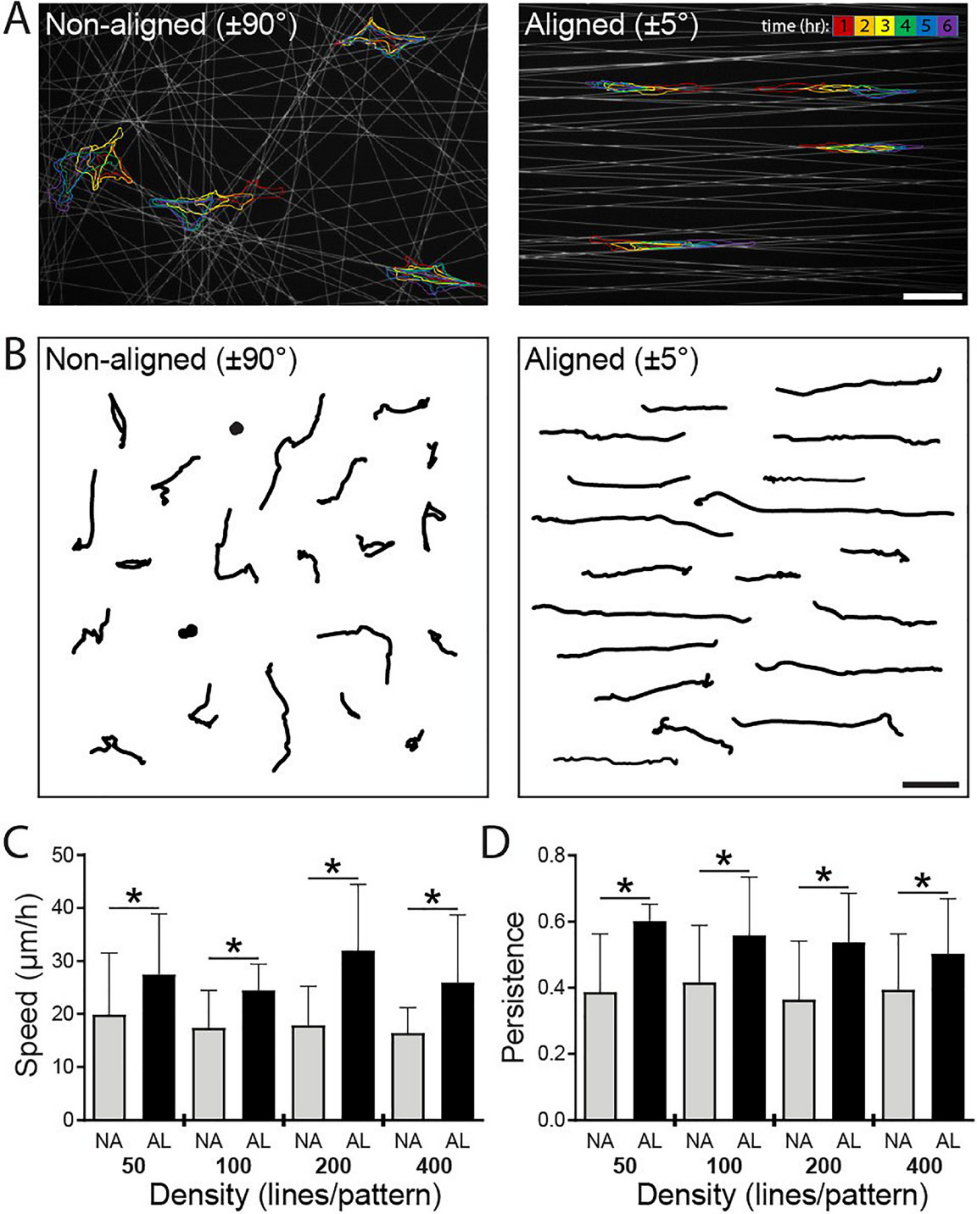
ECM alignment influences migration speed independent of ECM density. (a) Overlaid outlines of representative cells at the end of each hour (as denoted by color bar) over 6 h time-lapse imaging on non-aligned (NA, ±90°) and aligned (AL, ±5°) Fn-patterns (grey) containing 100 lines with a width of 1 *μ*m. (b) 20 representative cell migration tracks measured over 6 h duration of time-lapse imaging on NA and AL patterns containing 100 lines with a width of 1 *μ*m. Average migration speeds (c) and persistence (d) on NA and AL patterns containing 50, 100, 200, or 400 lines (n ≥ 10 cells per condition, ≥160 cells analyzed total, * indicates a significant difference with p < 0.05 vs. NA group at the same line density). Scale bars: 100 *μ*m.

Previous work has linked an elongated, uniaxial cell shape with increased migration efficiency,^20^ and so, we next examined the functional consequence of ECM organization on migration speed and persistence using our two extremes of alignment: aligned (±5°) and non-aligned (±90°) patterns (supplementary material, Fig. 1). We performed live time-lapse imaging over 6 h, utilizing Hoechst-labelled nuclei to track cell movement with an automated image analysis algorithm. Representative temporal overlays of the cell shape on non-aligned patterns reveal a dynamic cell shape, while cells on aligned patterns maintained an elongated morphology throughout their migration track [Fig. 4(a)]. Aligned patterns also produced more directionally persistent migration and faster speeds compared to cells migrating on non-aligned patterns, and furthermore, this effect remained consistently true over the full range of line densities examined [Figs. 4(b)–4(d)]. Tracking HT1080 fibrosarcoma cell migration, we similarly found that aligned patterns produced faster speeds and more directionally persistent migration compared to non-aligned patterns across the full range of line densities examined (supplementary material, Fig. 2). These results parallel reports of increased cell migration efficiency on aligned collagen gels^10,59^ and topographically patterned substrates,^22,60,61^ supporting the conclusion that a high degree of ECM anisotropy critically defines a uniaxial phenotype and directed cell migration. While many studies examining migration on aligned ECM report enhanced persistence and directionality, net migration speed has not consistently been observed to increase with directed migration. Our finding of increased speeds may stem from the comparison of non-aligned vs. aligned substrates with comparable underlying adhesive areas or the fact that proteolytic activity is dispensable for migration in this setting.

### ECM alignment polarizes focal adhesions and localizes Rac activity to stabilize active protrusions

**FIG. 5.**
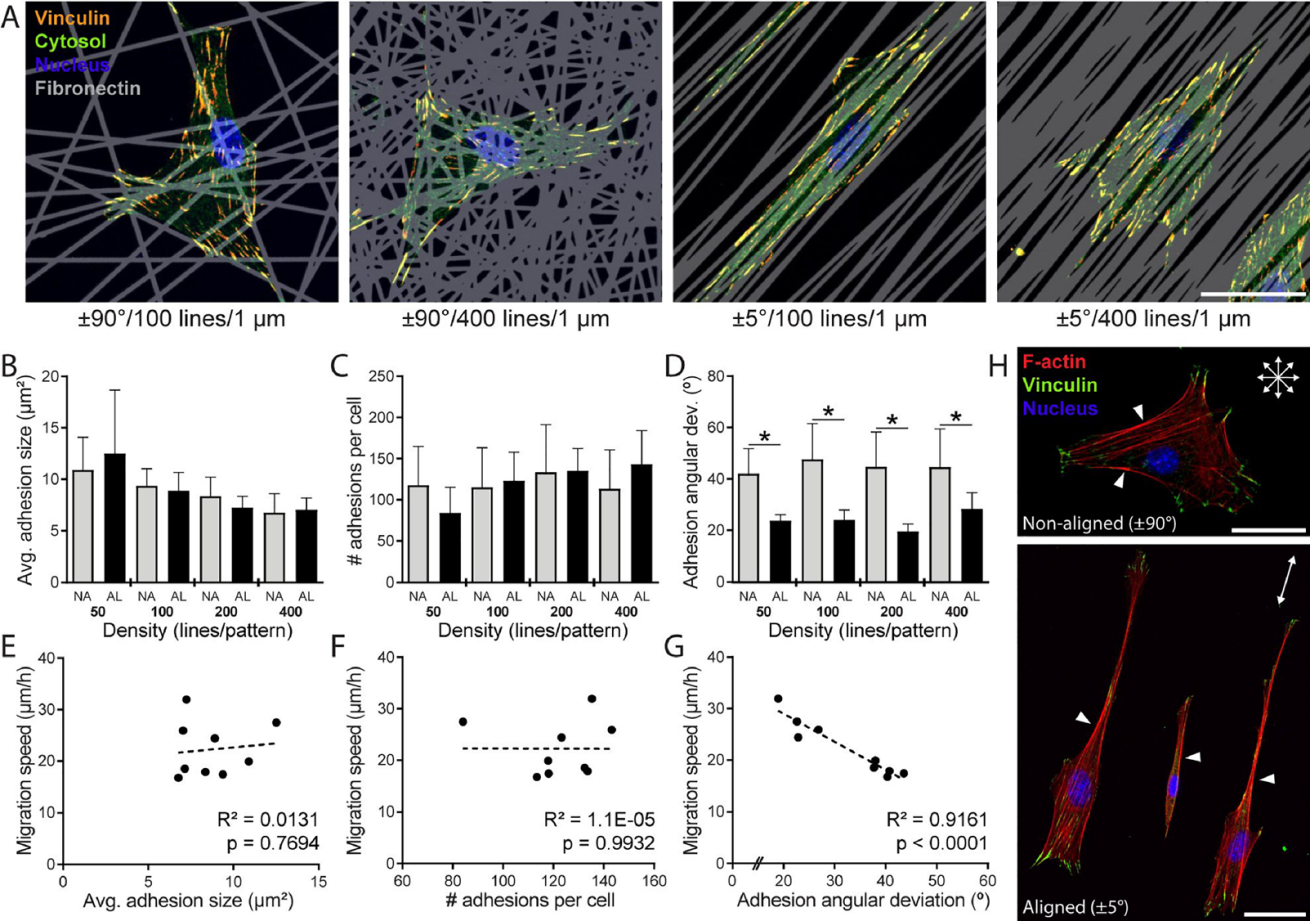
Focal adhesion organization parallels the alignment of ECM patterns. (a) Representative confocal images of 3T3 fibroblasts on non-aligned (±90°) and aligned (±5°) patterns with 100 or 400 line elements stained for vinculin to localize focal adhesions (yellow: anti-vinculin, green: cytosol, blue: nucleus, and grey: Fn555; scale bar: 50 *μ*m). Average individual focal adhesion size (b), total number of adhesions per cell (c), and angular deviation of adhesion orientation within a given cell (d) for cells on non-aligned (NA, ±90°) and aligned (AL, ±5°) patterns containing 50, 100, 200, or 400 lines (n ≥ 9 cells analyzed per condition, total of 101 cells analyzed, * indicates a significant difference with p < 0.05 comparing AL vs. NA group at the same line density). (e)–(g) Correlations of average migration speed vs. average adhesion size (e), total number of adhesions per cell (f), and the angular deviation of FA orientation (g). Each data point represents the population average taken from a different pattern. Dashed lines indicate the linear regression lines, with R^2^ and p-values indicated within each plot. (h) Representative confocal images of fibroblasts on NA and AL patterns with 200 line elements stained for vinculin and F-actin (green: anti-vinculin, red: F-actin, and blue: nucleus; scale bars: 50 *μ*m).

During mesenchymal migration, cells form adhesions to the underlying ECM to exert the necessary traction forces required to advance the cell body forward.^62–67^ Looking for differences in morphometric features of focal adhesions (FAs) that could explain the observed differences in migration speed as a function of ECM alignment, we performed immunofluorescence staining against vinculin (a well-accepted marker of force bearing FAs),^68–70^ high resolution confocal imaging, and image analysis to quantify characteristics of FAs on aligned (±5°) and non-aligned (±90°) patterns over a range of ECM densities [Fig. 5(a), supplementary material, Fig. 1]. Examining the average adhesion size and the number of adhesions per cell, we found no consistent differences as a function of alignment [Figs. 5(b) and 5(c)]. However, when we examined the angular dispersion of adhesions with respect to the major axis of the cell, a significant discrepancy in adhesion organization existed between aligned and non-aligned ECM at each level of density examined [Fig. 5(d)]. Non-aligned ECM resulted in a population of adhesions with high angular dispersion with respect to each other; in contrast, aligned ECM promoted the organization of FAs along the cell's long axis [Fig. 5(a)]. This difference in adhesion angular deviation mirrored differences in migration speed across multiple ECM densities and proved to be significantly linearly correlated with an R^2^ value of 0.916 [Fig. 5(g), p < 0.0001]. Correlations between the other adhesion metrics quantified and migration speed proved to be non-significant [Figs. 5(e) and 5(f)]. These findings were additionally confirmed with HT1080 fibrosarcoma cells (supplementary material, Fig. 3). The observation that the global orientation of adhesions uniquely predicts migration speed is in contrast to recent reports highlighting FA size as a critical predictor. However, in contrast to the work at hand, these studies were performed on flat unpatterned substrates of varying stiffness, highlighting again a clear distinction between aligned uniaxial cell migration and unconstrained 2D migration.^71^

F-actin organization reflected the organization of FAs, where cells on non-aligned ECM with randomly oriented adhesions also had disorganized F-actin stress fibers [Fig. 5(h), top]. In contrast, uniaxial cells on aligned ECM possessed highly aligned stress fibers running predominantly in the direction of the long axis of the elongated cell body, mirroring the co-alignment of FAs [Fig. 5(h), bottom]. Alignment of FAs and F-actin bundles supports observations that FA sites facilitate actin stress fiber assembly^72,73^ and intracellular force transmission to the ECM occurs via actin engagement with FA proteins.^63,74,75^ For cells on aligned ECMs, we observed intense F-actin staining at the cell walls running along the direction of matrix alignment and the putative direction of migration [Fig. 5(h), arrows]. Cells on non-aligned ECM also possessed intense F-actin bundles along straight edges of the cell's periphery. Regardless of cell shape and orientation, FAs and active lamellipodial protrusions were seldom observed at such locations possessing robust stress fibers, echoing previous observations employing cell-sized geometric micropatterns.^76,77^ Given that the formation of F-actin stress fibers has been associated with augmented RhoA activity,^78–81^ previous studies suggest a mutual exclusion between RhoA and Rac1 localization,^82–87^ and Rac1 activity is heavily implicated in protrusion activity,^2,79,88,89^ we hypothesized that highly elongated cells on aligned ECM would preferentially form protrusions at the distal ends of the cell in the direction of ECM alignment. Furthermore, we predicted that the stability of these protrusions would be enhanced compared to cells on disorganized (non-aligned) ECM.

**FIG. 6.**
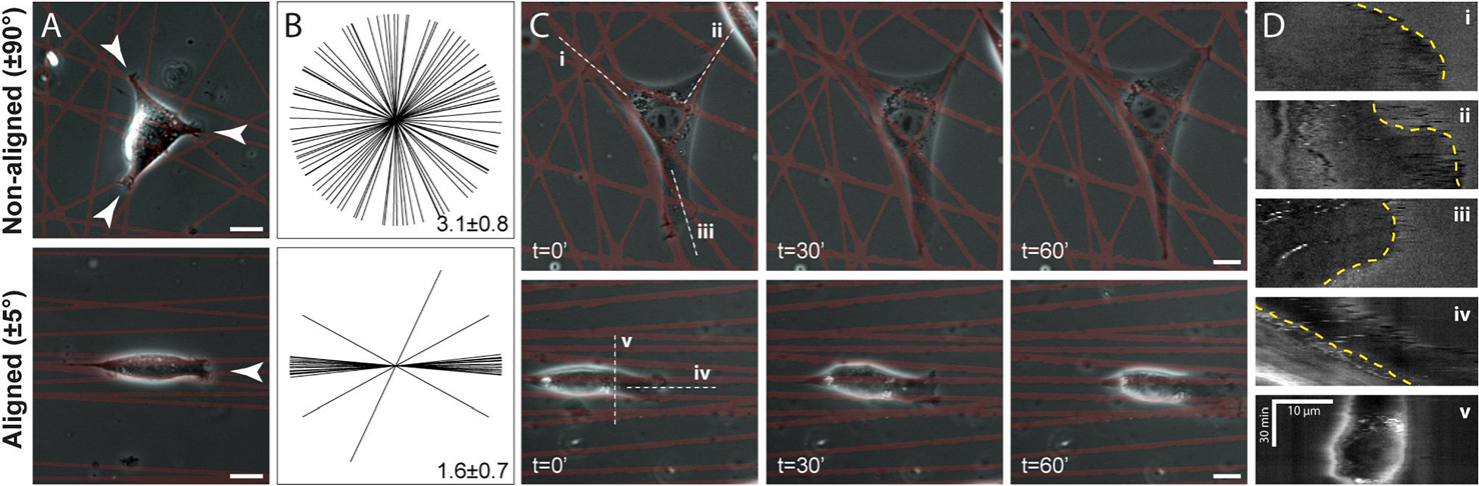
ECM alignment dictates the number and stability of active protrusions. (a) Representative phase images of 3T3s on aligned and non-aligned patterns containing 100 line elements. White arrows show locations of active protrusions (identified by ruffling activity via time-lapse imaging). (b) Starburst plots indicating the angle of active protrusions (n = 16 cells for each condition) and average number of protrusions per cell (mean ± standard deviation). (c) Phase images from time-lapse series at 0, 30, and 60 min. (d) Kymographs produced from locations indicated with white dashed lines in left-most images of C over 60 minute intervals. Leading or retracting edge indicated with yellow dashed lines (scale bars: 10 *μ*m).

By identifying ruffling edges in high resolution spatiotemporal time-lapse image series, we were able to determine the location and number of active protrusions on aligned (±5°) and non-aligned (±90°) patterns with 100 lines/pattern [Fig. 6(a), supplementary material, Fig. 1]. Indeed, ECM alignment resulted in a lower number of active protrusions per cell (1.56 ± 0.73 vs. 3.13 ± 0.81 on aligned vs. non-aligned, p < 0.0001), with the direction of 92% of all protrusions falling within ±45° of the direction of ECM alignment [Fig. 6(b)]. Furthermore, kymographs acquired at the location of ruffling protrusions highlighted a distinction in the stability of protrusions as a function of ECM organization [Figs. 6(c) and 6(d)]. On non-aligned ECM, different protrusions within the same cell were often observed to extend [Fig. 6(d)-i], pause for a period of time [Fig. 6(d)-ii], or retract [Fig. 6(d)-iii] concurrently. In contrast, aligned ECM promoted a steady extension of the cell's leading edge in the direction of ECM alignment [Fig. 6(d)-iv]. These observations along with the increase in migration speed on aligned ECM [Fig. 4(c)] support the notion that the maintenance of a single location of protrusive activity underlies efficient migration^20,90^ and clearly demonstrate that ECM organization dictates protrusive activity.

**FIG. 7.**
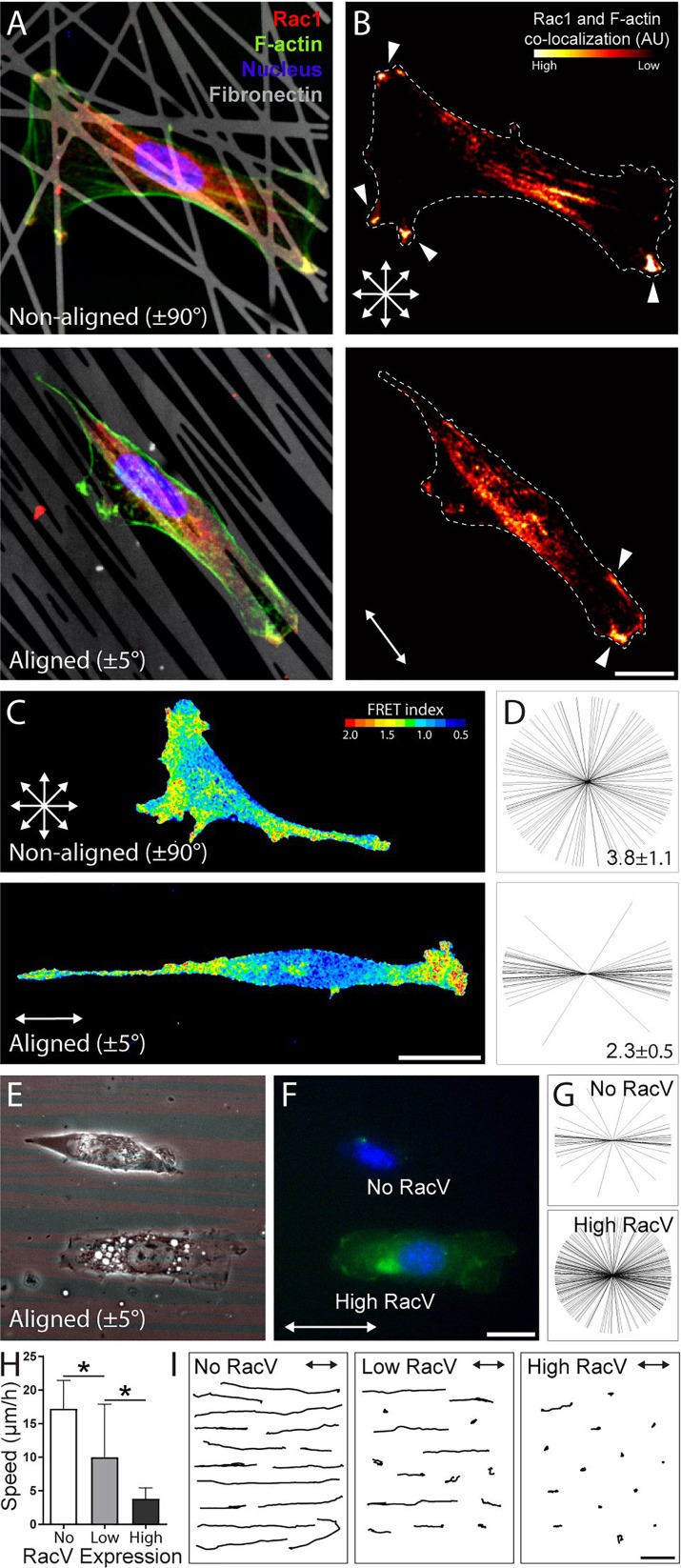
ECM alignment dictates the localization of Rac1-enriched extensions. (a) Representative confocal images and (b) corresponding heatmaps of 3T3s stained for active Rac on NA (top) and AL (bottom) patterns containing 100 lines with a width of 1 *μ*m [red: active Rac (PBD-GST, anti-GST antibody), green: F-actin, blue: nucleus, and grey: Fn555; scale bar: 25 *μ*m]. Heat maps indicate co-localization of Rac activity and F-actin. White arrows indicate the direction of pattern alignment (scale bar: 25 *μ*m). (c) Maximum projections of confocal stacks of live-3T3 migration expressing a Rac1-PBD FRET biosensor. Pseudocolored intensity scales indicating that FRET activity was maintained for each condition; scale bars, 25 *μ*m. (d) Starburst plots indicating the angle of Rac1-enriched protrusions on NA (top) and AL (bottom) patterns (n ≥ 15 cells for each condition) and the average number of active Rac1 protrusions per cell (mean ± standard deviation). (e) Corresponding phase and (f) fluorescence micrograph of representative transfected 3T3s that highly expressed (“High RacV,” green, bottom) and failed to express (“No RacV,” no fluorescence, top) the RacV12-GFP construct (red: Fn555, green: GFP, scale bar: 25 *μ*m). (g) Starburst plots indicating the angle of active protrusions for non- and high expressers of the RacV construct (n ≥ 16 for each condition). (h) Migration speed as a function of RacV expression levels; cells were grouped into three expression levels: no, low, and high (n ≥ 15 for each condition, * indicates a significant difference with p < 0.05). (i) 15 representative cell migration tracks of 3T3s with no, low, and high RacV expression measured over 6 h duration on aligned patterns containing 100 lines (scale bar: 100 *μ*m).

Given these striking distinctions in the location of active protrusions and Rac1's known role in driving lamellipodial protrusion,^90,91^ we next examined whether ECM alignment also altered the localization of active Rac1 signaling. Glutathione-S-Transferase (GST)-tagged protein binding domain (PBD) and immunofluorescence staining were used to identify intracellular locations of enriched active Rac1 on aligned (±5°) and non-aligned (±90°) patterns with 100 lines/pattern [Fig. 7(a), supplementary material, Fig. 1]. Cross-correlating fluorescence intensities from Rac1 and F-actin images allowed us to identify locations of high co-localization of these two proteins, both critical to protrusion activity [Fig. 7(b)]. Qualitatively, we observed that cells on non-aligned ECM possessed multiple protrusions with co-localization of these two signals. Conversely, cells on aligned ECM possess fewer protrusions with hot spots, and these protrusions were additionally in the direction of ECM alignment and putative migration. These observations suggest that ECM alignment influences the number and location of active protrusions, contributing to the stable and directional protrusions underlying uniaxial directional migration. To further support these observations, we employed a Förster resonance energy transfer (FRET)-based approach to image Rac1 activity modified from a previously reported construct. Within this single chain bioactivity reporter, FRET between molecules occurs upon binding of PBD to Rac1. Live imaging using this reporter revealed similar localization of Rac1 activity as a function of ECM alignment, with preferential Rac1 activation in directions corresponding to the underlying pattern alignment [Figs. 7(c) and 7(d)].

We next transiently transfected 3T3s with a green fluorescent protein (GFP)-tagged constitutively active mutant copy of Rac1 (RacV12-GFP, abbreviated RacV) and plated cells onto aligned ECM (±5°, 200 lines/pattern, 1 *μ*m width). As expression of the plasmid was heterogeneous, we segregated transfected cells into three distinct populations based on GFP intensity: no, low, and high expression [Figs. 7(e) and 7(f)]. High RacV expression resulted in an increased frequency of active protrusions off-axis to the direction of ECM alignment relative to non-expressing cells [25% vs. 8%, Fig. 7(g)]. Strikingly, the loss of protrusion directionality with increasing RacV expression paralleled decreases in migration speed where the highest expression of RacV resulted in active protrusions along the entire cell periphery and virtually no cell movement [Figs. 7(h)–7(i)]. These findings build on previous work by Pankov *et al.* showing how graded levels of Rac1 activity switch cells between directed and random migration on unpatterned 2D substrates.^90^ In addition to total levels, it is now clear that localization of active Rho GTPase are critical to cell polarity and subsequent movement.^87,92^ Our findings suggest that ECM alignment and therefore anisotropy of the local adhesive microenvironment can modulate cell shape and cytoskeletal architecture to spatially define the location of such signals.

### Summary and outlook

Many engineered models such as 1D patterned lines, nanopatterned ridges and grooves, and electrospun fibers have been utilized to understand how contact guidance cues provided by fibrous ECM influence cell migration.^20,23,60,93^ Previous studies primarily compared aligned, adhesion-restrictive substrates versus uniformly coated ECM substrates, resulting in differences in ECM anisotropy as well as total adhesive area. In contrast, this work provides a microcontact printing-based platform modeling the ECM as collections of fibronectin lines with graded variations of alignment, density, and width to identify how combinations of these parameters influenced cell migration. With high content imaging and statistical analyses, we identified matrix alignment as a critical parameter in influencing cell morphology, polarization, and migratory behavior. Interestingly, we find that cells on highly aligned ECM possess organized FAs. This alignment of FAs correlates strongly with directional, high speed migration in stark contrast to other morphometric features of FAs. Highly aligned FAs and associated F-actin stress fibers result in the formation and stabilization of cell protrusions in the direction of ECM alignment, and our data suggest that this control occurs through the localization of active Rac1. This work clearly indicated that anisotropy of the adhesive ECM, independent of substrate dimensionality, dictates the cytoskeletal architecture required for directed cell migration. Furthermore, these micropatterns could provide a high-throughput screen of ECM parameters on other cell functions and may aid in identifying novel therapeutics that selectively inhibit directionally invasive tumor cells.

## METHODS

### Cell culture and biological reagents

NIH3T3 fibroblasts were cultured in high glucose Dulbecco's Modified Eagle Medium containing 1% penicillin/streptomycin, L-glutamine (ThermoFisher Scientific, Waltham, MA), and 10% bovine serum (Atlanta Biologicals, Flowery Branch, GA). Cells were passaged upon achieving confluency at a ratio of 1:4 and used for studies until passage 20. For all studies, cells were trypsinized, counted, and seeded onto substrates at a density of 2000 cells/cm^2^. For overexpression studies, EGFP-RacV12, a constitutively active mutant (valine substitution at amino acid residue 12) of Rac1 fused to enhanced green fluorescent protein (gift from M. Philips, NY University Medical Center, New York, NY), was transiently transfected using Lipofectamine 2000 (Life Technologies) a day before seeding onto microcontact-printed substrates.

### Photolithography and microcontact printing

To produce microcontact-printed ECM screening substrates that model the spatial patterning of adhesive ligands in the fibrous extracellular matrix, a Matlab (Mathworks, Cambridge, MA) script was written to generate patterns consisting of lines of with varying line widths (2.5, 3.5, 5, and 10 *μ*m), densities (50, 100, 200, 400, and 800 lines per field), and angular dispersions [±5° (most aligned), ±15°, ±30°, ±45°, ±60°, and ±90° (non-aligned or random)]. These angles reflect bounds between which each line angle was selected from a uniform distribution. Individual fields were exported in vector format, assembled *en mass* in Autocad (Autodesk, Mill Valley, CA) into arrays, and printed in chrome on a quartz reticle (Advance Reproductions, North Andover, MA). Using a Nikon G4 stepper enabling 5× optical reduction in line widths (0.5, 0.7, 1.0, and 2.0 *μ*m) (Penn Regional Nanotechnology Facility, Philadelphia, PA), patterns were transferred to a 5 in. silicon wafer spuncoat with 500 nm thickness Microposit S1813 photoresist (MicroChem, Westborough, MA). Following development, polydimethylsiloxane (PDMS) (Sylgard 184, Dow Corning, Midland, MI) was cast onto wafers and cured at 60 °C to generate micropatterned stamps.

To enable pattern visualization, fibronectin from human plasma (Fn, Corning, Corning, NY) was fluorescently tagged with AlexaFluor555 succidinyl ester following the manufacturer's protocol (Invitrogen, Carlsbad, CA). Briefly, a 1 mg/ml solution of fibronectin in 1 M sodium bicarbonate reacted with AlexaFluor555 succidinyl ester at a 9-fold molar excess for 2 h at RT with continual agitation. Unconjugated fluorophores were removed by overnight dialysis (6.5 kDa cutoff), and the concentration of conjugated fibronectin (Fn555) was determined by absorbance at 280 and 555 nm using a spectrophotometer. For microcontact printing, Fn555 was diluted to 50 *μ*g/ml in phosphate buffered saline (PBS) and adsorbed uniformly onto a 1.5 cm by 1.5 cm piece of flat PDMS cast from a cleaned silicon wafer. Patterned stamps containing the negative of the intended final features were activated via UV ozone and applied to the Fn-inked stamp to selectively remove background (non-feature) areas of Fn. The remaining patterned Fn was transferred to the final cell culture substrate by applying the inking stamp a UV ozone activated, PDMS coated coverslip (via spin coater, 5000 RPM). Following storage overnight to allow for recovery of hydrophobicity, substrates were incubated in Pluronics F-127 (0.2% w/v in deionized water, Sigma-Aldrich, St. Louis, MO) for 30 min at 25 °C to prevent non-intended protein adsorption and undesired cell adhesion to non-printed regions.

### Immunofluorescence

To examine cell morphology and the organization of the actin cytoskeleton, cells were fixed in 4% phosphate-buffered paraformaldehyde for 10 min and then permeabilized with 0.03% Triton X-100 for 10 min. Filamentous actin was stained with phalloidin-AlexaFluor488 (Life Technologies), and cell nuclei were stained with Hoechst33342, (1 *μ*g/ml, Sigma-Aldrich) blocked in 2% bovine serum albumin. For immunofluorescence staining, fixation and permeabilization were performed as above (unless specified otherwise) followed by incubation in blocking solution (10% fetal bovine serum in PBS) for 1 h, primary antibody (below) dilution in blocking solution for 1 h, three PBS washes for 5 min each, a 1:1000 dilution of AlexaFluor conjugated IgG antibody (Life Technologies) in blocking solution for 1 h, and two PBS washes for 5 min each. To stain the microtubule organizing center (MTOC), a 1:500 dilution of rabbit anti-pericentrin was employed as the primary antibody (PRB-432C, Covance). To stain focal adhesions, samples were permeabilized and fixed simultaneously and a 1:500 dilution of monoclonal mouse anti-vinculin antibody was employed as the primary antibody (V9264, Sigma-Aldrich). To stain for Rac activity, samples were incubated with glutathione-eluted protein binding domain (PBD)-GST overnight at 4 °C (Cytoskeleton), rinsed with PBS, and incubated with goat anti-GST antibody (27-4577-01, GE Healthcare). Imaging was performed on a Nikon Eclipse Ti (10×) or on a Zeiss 710 laser scanning microscope (40×), and images are presented as maximum intensity projections.

### Rac1 FRET imaging

To examine Rac1 activity, we modified a previously designed RaichuEV-Rac1 FRET biosensor^24^ by replacing the mTurquoise/YPet fluorophore pair with Clover/mRuby2 (RaichuEV-Rac1-CR) to reduce fluorophore bleed-through, improve the FRET dynamic range, and enable incorporation into lentiviral vectors. NIH3T3s were transiently transfected with the RaichuEV-Rac1-CR biosensor using Lipofectamine LTX with Plus Reagent (ThermoFisher Scientific) 24 h before seeding onto microcontact-printed substrates. Cells expressing RaichuEV-Rac1-CR were imaged 12 h after seeding. The binding of active Rac1 was detected by imaging the FRET-dependent, intramolecular emission fluorophore (mRuby2) from RaichuEV-Rac1-CR as previously described.^12^ Briefly, optimal FRET acquisition settings were determined for the Zeiss LSM 800 confocal microscope and strictly maintained during all subsequent FRET imaging; intensity levels of biosensor expression were similarly carefully controlled and maintained between selected cells. Images of mClover and mRuby2 were obtained for each z-plane under 488 nm and 567 nm illumination. Summed projections of confocal z-stacks were generated using ImageJ software. Images were first background subtracted, and a binary mask was applied by thresholding to the Acceptor (mRuby2) channel to isolate the cellular signal. Pixel-by-pixel FRET to Donor (Clover) ratio images were generated in ImageJ. All the resulting FRET ratio images were processed with a 3 × 3 median filter to remove any hot pixels and presented in a scaled 16 color lookup table (ImageJ).

### Microscopy and image analysis

For migration studies, sample media were supplemented with 1 *μ*g/ml Hoechst33342 and samples were incubated for 30 min to label cell nuclei. Coverslips were then transferred to Attofluor chambers (ThermoFisher Scientific) and media were refreshed. Samples were imaged every 10 min for a duration of 6 h on a Nikon Eclipse Ti epifluorescence microscope equipped with a custom-built environmental chamber (37 °C, 5% CO_2_). Following raw image export, nuclear tracking was performed with a custom Matlab script predicated on the IDL Particle Tracking code.^25^ Briefly, parameters to threshold and locate the centroids of cell nuclei were identified and applied uniformly across the entire dataset. Centroids of nuclei in serial images were linked using IDL to define migration tracks. Migration speed was calculated as the total tracked distance over the total tracking duration. Persistence was defined as the distance between initial and final positions normalized to the total tracked distance. Cells that underwent proliferation or were non-migratory over the tracked duration were not analyzed, except in Rac1 perturbation studies. Kymographs were generated in ImageJ.

Additional Matlab scripts were created to analyze cell morphology and focal adhesion characteristics. For cell morphometric data, images were acquired from 4′,6-diamidino-2-phenylindole/phalloidin-stained samples. For focal adhesion analysis, images were acquired from samples immunostained for vinculin. In both cases, images were imported, background filtered, and manually thresholded, with identical threshold values applied across entire image sets. Outlines of individual features were extracted, and shape characteristics (via regionprops) including the area, aspect ratio, and orientation were exported.

### Statistics

Significance was determined by one-way analysis of variance (ANOVA) with Bonferroni post hoc tests and generally established with p < 0.05, unless specified otherwise. For cell and adhesion orientation data, angular means were determined using circular statistics in Matlab (circstat). Correlation between variables was tested using Pearson's product-moment correlation and the correspondent test.^26,27^ Multinomial multivariate linear regression model building was performed using a full linear regression model, and backward selection was performed using F-test p-values. Comparisons between models of different sizes were performed using the Aikake Information Criteria.^28^ Differences within variability between groups were tested using Levene's test. Analysis was performed using GraphPad Prism 6 or program R (v3.1.0).

### Ethics approval

No ethics approval was required for the experiments described in this study.

## SUPPLEMENTARY MATERIAL

See supplementary material for a substrate map depicting the patterns utilized in Figs. 1–7 and additional cell migration and focal adhesion data utilizing HT1080 fibrosarcoma cells.
